# Best practice integrated primary/secondary health care governance - applying evidence to Australia’s health reform agenda

**DOI:** 10.1186/1472-6963-14-S2-O6

**Published:** 2014-07-07

**Authors:** Caroline Nicholson, Claire Jackson, John Marley

**Affiliations:** 1 University of Queensland, Brisbane, Queensland, Australia; 2 Mater Health Services, South Brisbane, Queensland, Australia

## Background

There is an identified need for more robust and high-quality evidence to inform decisions about how to develop and deliver integrated primary/secondary health care. There is no single model of integrated care that is suited to all contexts, settings and circumstances. Researchers and policy-makers need to work together with practitioners to develop, evaluate and implement effective approaches. For the goals of health reform to be realized, primary health care and secondary care organisations must work together to achieve co-ordinated and integrated healthcare services. This study aimed to describe the elements of a health care system capable of supporting effective integrated primary/secondary care and how many of these governance elements are identifiable within Australia’s current health care reform environment.

## Materials and methods

This study presents the results of a systematic review in the development of a framework to achieve a ‘best practice’ governance model for integrated primary/secondary health care [1] and the application of the findings to key policy statements regarding integrated care delivery [2].

## Results

The systematic review identifies ten elements linked to successful primary/secondary health care integration projects - a population focus; shared clinical priorities; joint planning; using data as a quality improvement tool across the continuum; innovation; effective change management; an appropriately trained workforce; integrated information communication systems; incentives; and, patient engagement. The Australian reform environment has made steady progress in building integrated governance arrangements around some elements, whilst others remain ad-hoc or non-existent. Formal documents mostly relate to silos of sector activity and not the interface.

## Conclusions

To apply important evidence to health care policy, and maximise reform success, we must review current governance frameworks to address the gaps identified in this paper. Whilst it is challenging to bring historically-disparate partners together into formal agreements, they are essential to creating the scalable ‘business rules’ and sustainable environment required to achieve the new care models we seek.
